# Outcome of artemether-lumefantrine treatment for uncomplicated malaria in HIV-infected adult patients on anti-retroviral therapy

**DOI:** 10.1186/1475-2875-13-205

**Published:** 2014-05-30

**Authors:** Betty A Maganda, Omary MS Minzi, Appolinary AR Kamuhabwa, Billy Ngasala, Philip G Sasi

**Affiliations:** 1 Department of Pharmaceutics, School of Pharmacy, Muhimbili University of Health and Allied Sciences, P.O. BOX 65013, Dar es Salaam, Tanzania; 2 Unit of Pharmacology and Therapeutics, School of Pharmacy, Muhimbili University of Health and Allied Sciences, P.O. BOX 65013, Dar es Salaam, Tanzania; 3 Department of Parasitology, School of Medicine, Muhimbili University of Health and Allied Sciences, P.O. BOX 65012, Dar es Salaam, Tanzania; 4 Department of Clinical Pharmacology, School of Medicine, Muhimbili University of Health and Allied Sciences, P.O. BOX 65015, Dar es Salaam, Tanzania

**Keywords:** Efavirenz, Nevirapine, Artemether-lumefantrine, Malaria treatment outcome

## Abstract

**Background:**

Malaria and HIV infections are both highly prevalent in sub-Saharan Africa, with HIV-infected patients being at higher risks of acquiring malaria. The majority of antiretroviral (ART) and anti-malarial drugs are metabolized by the CYP450 system, creating a chance of drug-drug interaction upon co-administration. Limited data are available on the effectiveness of the artemether-lumefantrine combination (AL) when co-administered with non-nucleoside reverse transcriptase inhibitors (NNRTIs). The aim of this study was to compare anti-malarial treatment responses between HIV-1 infected patients on either nevirapine- or efavirenz-based treatment and those not yet on ART (control-arm) with uncomplicated falciparum malaria, treated with AL.

**Method:**

This was a prospective, non-randomized, open-label study conducted in Bagamoyo district, with three arms of HIV-infected adults: efavirenz-based treatment arm (EFV-arm) n = 66, nevirapine-based treatment arm (NVP-arm) n = 128, and control-arm n = 75, with uncomplicated malaria. All patients were treated with AL and followed up for 28 days. The primary outcome measure was an adequate clinical and parasitological response (ACPR) after treatment with AL by day 28.

**Results:**

Day 28 ACPR was 97.6%, 82.5% and 94.5% for the NVP-arm, EFV-arm and control-arm, respectively. No early treatment or late parasitological failure was reported. The cumulative risk of recurrent parasitaemia was >19-fold higher in the EFV-arm than in the control-arm (Hazard ratio [HR], 19.11 [95% confidence interval {CI}, 10.5–34.5]; P < 0.01). The cumulative risk of recurrent parasitaemia in the NVP-arm was not significantly higher than in the control-arm ([HR], 2.44 [95% {CI}, 0.79–7.6]; P = 0.53). The median (IQR) day 7 plasma concentrations of lumefantrine for the three arms were: 1,125 ng/m (638.8-1913), 300.4 ng/ml (220.8-343.1) and 970 ng/ml (562.1-1729) for the NVP-arm, the EFV-arm and the control-arm, respectively (P < 0.001). In all three arms, the reported adverse events were mostly mild.

**Conclusion:**

After 28 days of follow-up, AL was statistically safe and effective in the treatment of uncomplicated malaria in the NVP-arm. The results of this study also provide an indication of the possible impact of EFV on the performance of AL and the likelihood of it affecting uncomplicated falciparum malaria treatment outcome.

## Background

Malaria and HIV-1 are the most common infections in sub-Saharan Africa and to a lesser extent in other developing countries [1]. It is estimated that about 38 million Africans are infected with HIV-1 and about 300–500 million people suffer from malaria each year [2,3]. Worldwide, the two diseases cause more than four million deaths per year [1]. HIV infection has been associated with an increase in malaria parasite density, delayed parasite clearance, a higher incidence of clinical and severe malaria, and death [4-12]. Diminished immunity of the host due to HIV and malaria co-infection may result in malaria impaired treatment response and an increased risk of recrudescence and re-infections [6-8,13-20].

The World Health Organization (WHO) recommends the use of artemisinin-based combination therapy (ACT) as first-line treatment for uncomplicated falciparum malaria in all malaria-endemic countries [21]. Artemether-lumefantrine (AL) is one of the most widely used ACT in malaria-endemic countries for the treatment of uncomplicated falciparum malaria, including Tanzania [21]. On the other hand, triple antiretroviral therapy (ART) is recommended for the management of HIV infection. The complex pharmacology of both ACT and antiretroviral drugs (ART) creates concerns about the safety and effectiveness of these agents when used simultaneously [22].

Artemether is metabolized to dihydroartemisinin (DHA) via cytochrome P450 (CYP) CYP3A4, CYP2B6 and possibly CYP2A6 [23]. Lumefantrine is metabolized by N-debutylation, mainly by CYP3A4, to desbutyl-lumefantrine [23-26]. Nevirapine (NVP) and efavirenz are non-nucleoside reverse transcriptase inhibitors (NNRTI), and are components of most first-line ART regimens in sub-Saharan Africa. These drugs are metabolized via CYP3A4 and CYP2B6 and induce their own metabolism via induction of CYP3A4 and 2B6 [27-30]. The involvement of almost the same isoenzymes in which some are inhibited and induced while other substances act as substrates or auto-inducers creates the potential for drug-drug interactions (DDIs) during co-administration of NNRTI and AL. Unfavourable DDIs may lead to supra-therapeutic concentrations due to enzyme inhibition resulting in toxicity or, conversely, sub-therapeutic concentrations resulting in treatment failure or drug resistance [22]. There may also be a beneficial interaction leading to positive pharmacological response in those cases where the parent compound is active and less metabolized.

The treatment guidelines for uncomplicated malaria set by the National Malaria Control Programmes in sub-Saharan Africa do not discriminate between the dosing regimen of AL in non-HIV infected patients and those undergoing ART.

To date, most of the reported studies describe the effect of HIV-1 infection on malaria treatment outcome in the absence of antiretroviral drug treatment [4-7,12-20]. The potential for DDIs between antiretroviral drugs (ART) and ACT in HIV-1 infected patients without malaria infection has been described in a few studies [31-34]. To date, there is inadequate information on malaria treatment outcome in HIV-1 infected patients on ART. This study reports the treatment outcomes of uncomplicated falciparum malaria in HIV-1 infected patients on ART and those not yet on ART.

## Methods

### Study site and ethical approval

This study was conducted between May 2010 and August 2012 at an HIV clinic at Bagamoyo District Hospital. Bagamoyo district is an area of moderate malaria transmission. In this district, the peak time for malaria transmission peak time is usually around April to August. The study was approved by the Muhimbili University of Health and Allied Sciences (MUHAS) research and ethics committee and was conducted according to Good Clinical Practice. To ensure confidentiality, patients were identified by special identification numbers.

### Study population

This study involved HIV-1 infected adults receiving ART as 200 mg NVP twice daily or 600 mg EFV at night for more than two months and those not yet on ART. Enrolled in the study were 128 patients in the nevirapine-based treatment arm (NVP-arm), 66 patients in the efavirenz-based treatment arm (EFV-arm) and 75 patients not yet on ART (control-arm).

### Patients’ eligibility and enrollment

All HIV-1 infected patients presenting at the HIV clinic for routine medical care, with fever and other symptoms suggestive of uncomplicated falciparum malaria such as chills, sweats, headaches, muscle aches, nausea, vomiting, diarrhoea, body weakness, poor appetite, pallor and enlarged spleen were screened for eligibility. Patients were only enrolled into the study after meeting the inclusion criteria: Aged ≥ 18 years; reported fever within the last 24 hours and/or an axillary temperature ≥ 37.5°C and with any of the above-mentioned symptoms of uncomplicated falciparum malaria; haemoglobin ≥ 7 g/dl and ≥ 35 kg body weight; microscopically-confirmed *Plasmodium falciparum* with no signs of complicated (severe) malaria; no history of an allergic reaction or serious side effects to AL or treatment with anti-malarial drugs for at least four weeks prior to enrollment; no evidence of chronic diseases, such as renal or liver failure; not on anti-tuberculosis drugs for at least three months prior to enrollment; not pregnant or a nursing mother; easy accessibility to the health-care facility (travel time < 2 hours) and willingness to attend for the stipulated follow-up visits. Before enrollment, written informed consent was obtained from all patients. All patient information was recorded in a case report form (CRF). The enrolled patients were encouraged to take their ART and AL as prescribed.

### Study design, treatment and procedures

This was a prospective, non-randomized, open-label, parallel and three-arm study. Patients were followed up for 28 days. Patients meeting the inclusion criteria were enrolled and took the full dose (three-day course) of AL (Coartem® containing 80 mg of artemether and 480 mg of lumefantrine, Novartis-Basel, Switzerland) at 0, 8, 24, 36, 48 and 60 hrs. The first and fifth doses of AL were taken by direct observed therapy (DOT) with 250 ml of milk (3.5% fat). The other four doses were taken at home. All patients were given verbal instructions on dosing intervals and on the importance of combining treatment with fatty meals. Additionally, patients were supplied with 10 extra 250 ml packets of milk (3.5% fat) to be taken with the rest of the doses at home. For the first and fifth doses, which were given by DOT and paracetamol was administered to all febrile patients.

Patients involved in this study were counseled to abstain from using alcohol, tea, caffeine and any drugs which may induce CYP3A4, such as griseofulvin, prednisolone, phenytoin, carbamezapine and phernobarbital. Non-prescription drugs, herbal medicines, oral contraceptives, grapefruits or grapefruit juice were also prohibited during the study.

At enrollment all patients gave a finger-prick blood sample for thick smear and for haemoglobin (Hb) estimation. Blood slides for malaria parasites were all read at Ifakara Health Institute-Bagamoyo Research and Training Centre (IHI-BRTC). Venous blood was collected at pre-determined times for quantification of lumefantrine plasma concentrations. Patients’ baseline CD4+ cell count was obtained from their records, the timeline being within 3 months prior to study enrollment.

Laboratory and clinical assessments were conducted on days 2, 3, 7, 14, 21 and 28 or on any day of recurrent illness. A reminder was sent to all patients by a study nurse via telephone about their medication and study visit schedules. Patients were encouraged to return to the study site any time they felt ill. Patients who failed to return on the scheduled day were visited and assessed at home. If the study nurse failed to locate a patient’s house, they were classified as lost to follow-up. Any additional medications taken during the study period were documented in the CRF.

The time to recurrent parasitaemia or the risk of recurrent parasitaemia (RP) was defined as the number of days between taking the first dose of AL and the day of microscopically detecting malaria parasites in the thick blood film. The time at risk ended whenever one of the following conditions occurred: RP, loss to follow-up, withdrawal, or end of follow-up period [35].

Patients with microscopically-confirmed *P. falciparum* during the 28 days of follow-up were treated with either quinine tablets or injection as described in the malaria treatment guidelines (2006) of Tanzania [36].

### Laboratory procedures

#### Microscopy

All thick blood smears were stained with 10% Giemsa stain for 30 minutes. Parasite density was estimated by counting the number of asexual parasites per 200 (per 1,000 for gametocytes) white blood cells (WBC) on a thick smear. Parasite density per μl was calculated by assuming a WBC count of 8,000 per μl [37]. All thick blood smears were independently read by two experienced microscopists. A smear was declared negative if no asexual parasites were seen after examining 200 high-power fields. An additional reading was performed for discordant results.

#### Blood sample collection and determination of lumefantrine concentrations

Blood samples from patients (4 ml) were collected in heparinized vacutainer tubes and centrifuged (×2000 g for 10 min) immediately to obtain plasma. Aliquots of plasma were transferred into 1 ml cryo-tubes, and stored at −80°C at the Ifakara Health Institute–BRTC until transfer to MUHAS for analysis. All patients’ plasma samples were analyzed at the MUHAS-Sida bio-analytical laboratory in Dar es Salaam. Lumefantrine concentrations were quantified using an HPLC method with UV detection as previously reported [38]. The coefficients of variation (CV%) during the analysis of lumefantrine were 2.5, 4.2 and 1.8% at 100, 1000, and 8,000 ng/ml, respectively. The lower limit of quantification was 50 ng/ml.

### Outcome measures

An adequate clinical and parasitological response in patients at 28 days after anti-malarial treatment was the primary study objective. The WHO guidelines for assessment and monitoring of anti-malarial drug efficacy for the treatment of uncomplicated falciparum malaria were used in the evaluation of the time to RP after treatment with AL [39]. Accordingly, the classification of treatment outcome was based on these guidelines. Adequate clinical and parasitological response (ACPR) was defined as the absence of parasitaemia by day 28 after initial treatment irrespective of axillary temperature, and not meeting any previous criteria for early treatment failure (ETF), late clinical failure (LCF), or late parasitological failure (LPF). The secondary outcome was day 7 lumefantrine plasma concentrations and the safety endpoint, which included clinical and laboratory adverse events.

### Statistical analysis

This study was considered to be exploratory. In total, 269 HIV-1 infected adult patients with uncomplicated falciparum malaria were enrolled with a minimum sample size of at least 50 patients for each arm [39]. The data was double-entered into a Microsoft access database, verified and exported to SPSS (version 16.0) software. The intention-to-treat approach was used to analyze the anti-malarial treatment response. The cumulative risk of RP was estimated using the Kaplan-Meier product limit formula and data were censored. Categorical variables were compared using the chi-square test. Descriptive statistics were used where appropriate. Continuous variables were compared using the one-way ANOVA test. Data is presented as frequencies, medians and means. A two-tailed P value < 0.05 was considered statistically significant.

## Results

### Study population baseline

**Figure 1:**
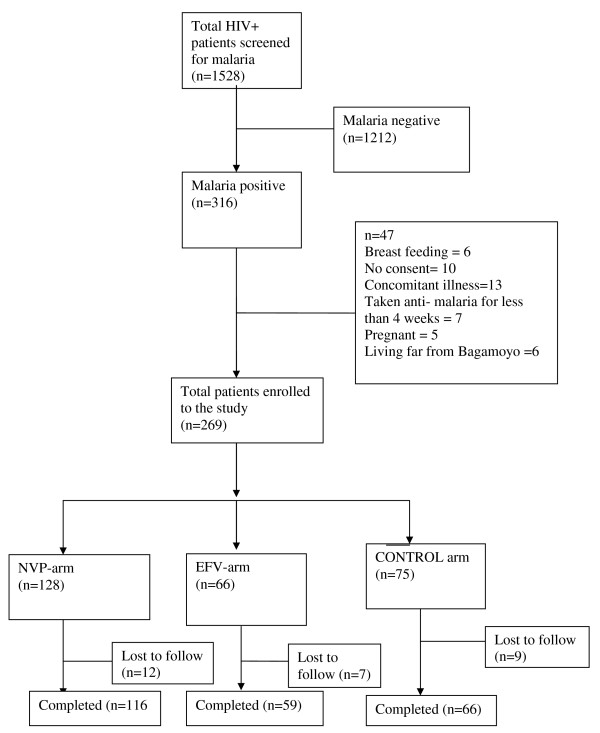
Study profile.

A total of 1,528 patients presenting at the HIV clinic with fever and other symptoms suggestive of malaria infection were screened. Among the screened patients, 316 (21%) had positive thick blood smears for malaria parasite. In total 269 (85%) patients met the inclusion criteria and were enrolled (Figure 1). In the studied population, 85 (31.6%) patients were males and 184 (68.4%) were females. Baseline clinical and demographic data showed no significant difference between the three arms (Table 1). Among the enrolled patients, 91% had baseline parasite density of ≤ 2,000/μl and one patient had ≥260,000/μl. Despite the high baseline parasitaemia, this patient had no general danger signs indicating severe disease.

**Table 1:** Patient baseline characteristics

**Parameters**	**ARMS**			**P-value**
	**Control group**	**Patient on NVP**	**Patient on EFV**	
	**(n = 75)**	**(n = 128)**	**(n = 66)**	
Sex (female) %	65	79.5	52.3	
Median age in years	38 (19–64)	42 (21–67)	43 (39–66)	0.015
Temperature mean, SD ± °C	38.1 ± 0.8	37.8 ± 1.3	38.3 ± 0.9	0.485
Median Weight (IQR)	56 (41–92)	55 (41–78)	58 (36–84)	0.953
Geometric mean parasite density, parasites/μL, SD ±	1280 (560–4040)	4040 (600–261520)	3440 (480–126960)	0.564
Haemoglobin (g/dL) median (IQR)	13.9 (12.2-15.2)	12.1 (11.2-13.5)	12.3 (10.2-13.6)	0.036
CD4^+^ count (x10^6^/L) median (IQR)	402 (66–964)	354 (19–1781)	298 (9–694)	0.002

The frequency of fever during the whole period of follow up was higher in the EFV-arm than in the NVP-arm or the control-arm, but the differences were not statistically significant (P = 0.82).

In the current study, about 48% of the enrolled patients had CD4 cell counts of < 350 cells/μl. Patients with high parasitaemia had low CD4 cell counts and there was a strong association between CD4 cell counts and parasitaemia (P < 0.001).

### Treatment outcomes

**Figure 2:**
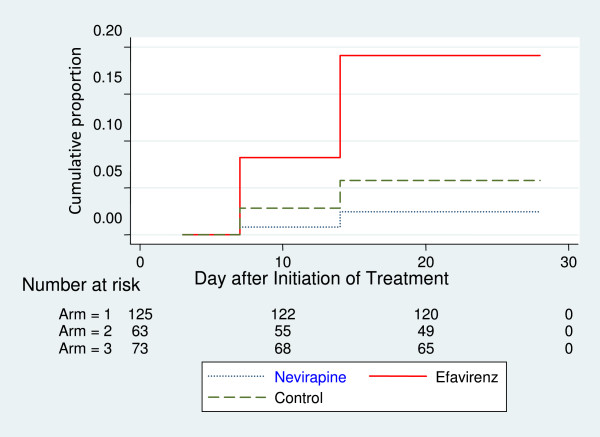
Kaplan Meier curve showing cumulative risk of treatment failure in HIV-1 infected patients treated with artemether-lumefantrine.

No early treatment failure or late parasitological failure was observed in the three arms. Overall, after day 28 of follow-up, 97.6% (95% CI, 92%–99%), 82.5% (95% CI, 70%–90%) and 94.5% (95% CI, 86%–98%) of patients in the NVP-arm, EFV-arm and control-arm, respectively, had no recurrent parasitaemia, thus meeting the WHO criteria for ACPR. The differences in the treatment outcome in the three arms were highly statistically significant (P < 0.001) (Table 2). The cumulative risk of recurrent parasitaemia on day 28 after initiation of treatment in the study population as a whole was about 7%. However, the cumulative risk of recurrent parasitaemia on day 28 after initiation of treatment was >19-fold higher in the EFV-arm than in the control-arm (Hazard ratio [HR], 19.11 [95% confidence interval {CI}, 10.5–34.5]; P < 0.01). Conversely, the cumulative risk of recurrent parasitaemia in the NVP-arm was not significantly higher than in the control-arm (Hazard ratio [HR], 2.44 [95% {CI}, 0.79–7.6]; P = 0.53) (Figure 2). In all three arms, there were no statistically significant differences in the risk of RP between patients with CD4 counts of > 350 cells/μl compared to those with CD4 counts of < 350 cells/μl (P = 0.204).

**Table 2:** Comparison of treatment outcomes among HIV-1 infected patients on ART and those not yet on an ART

**Treatment outcome**	**ARMS**		
	**Nevirapine (N = 125)**	**Efavirenz (N = 63)**	**Control (N = 73)**
ETF n, (%)	0	0	0
LTF n, (%)	3 (2.4)	11 (17.5)	4 (5.5)
LPF n, (%)	0	0	0
ACPR n, (%)	122 (97.6)	52 (82.5)	69 (94.5)
	RR = 0.4, 95% C.I = 0.29-0.9, P = 0.53	RR = 3.2, 95% CI, 2.4-7.8, P < 0.001	

### Day 7 lumefantrine plasma concentrations

**Figure 3:**
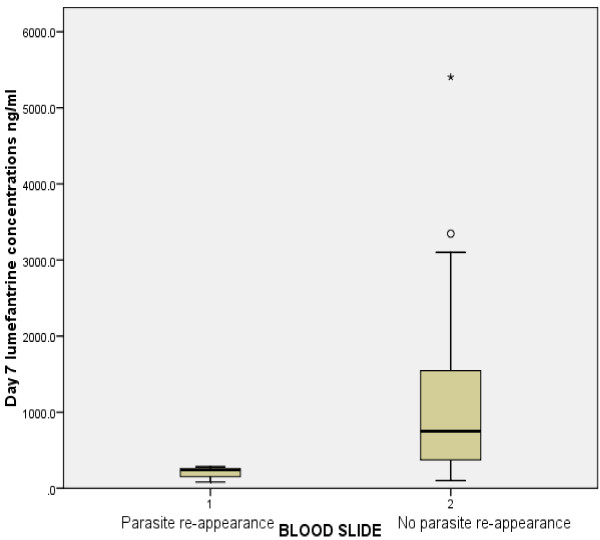
Box plots of day 7 lumefantrine plasma concentrations versus parasite re-appearance in all the three-arms.

In total, 251 (93%) patients had lumefantrine plasma concentrations measured on day 7 after initiation of treatment. The median (IQR) day 7 plasma concentrations of lumefantrine for the three arms were: 1,125 ng/ml (638.8-1913), 300.4 ng/ml (220.8-343.1) and 970 ng/ml (562.1-1729) for the NVP and EFV-based treatment arms and for the control-arm, respectively. The difference in day 7 lumefantrine plasma concentrations between the EFV-arm and the control-arm was statistically significant (P < 0.001), while there was no statistically significance difference in the day 7 lumefantrine concentration between the NVP-arm and the control-arm (P = 0.063). In all three arms, the median lumefantrine concentration was significantly lower in patients with RP as compared to those with no RP (Figures 3). Overall, 4% (3/69) of patients in the control-arm, 32% (19/60) in the EFV-arm and 3% (4/121) in the NVP-arm had lumefantrine concentrations of ≤ 280 ng/ml. Seven out of the eight patients with RP on day 7 had lumefantrine concentrations of < 280 ng/ml.

### Adverse events

AL was well tolerated in the study population. The most frequently reported adverse events were mild in severity; the frequencies of adverse events for the three arms are indicated in Table 3.

**Table 3:** Adverse events frequencies

**Adverse events**	**Arm**		
	**Nevirapine**	**Efavirenz**	**Control**
Palpitation (percentage)	23	15	12
Dizziness (percentage)	17	13	11
Severe headache (percentage)	4	3	2

## Discussion

In areas of high or moderate malaria transmission, response to malaria treatment mainly depends on the host’s immunity and the amount of drugs available in human plasma to clear the parasites. Patients with good immunity have the advantage of getting an adequate malaria cure with drug(s) as opposed to a subject with poor immunity. However, in patients with poor immunity, the initial rate of parasite clearance is determined by the intrinsic activity of the drug, the susceptibility of infecting parasites and the drug levels achieved [40]. The pattern of treatment failure is also determined by the above-mentioned factors.

Possible DDIs between ACT and NNRTIs in HIV-1 patients without malaria have been reported in a few studies [32-34]. This paper presents an evaluation of the effect of NNRTIs on anti-malarial treatment response in HIV-1 infected adult patients with uncomplicated falciparum malaria in an area of moderate transmission.

In the present study, all patients from the three arms were treated with artemether-lumefantrine and followed up for 28 days to determine malaria treatment response (ACPR). Based on WHO recommendations, the cure rates for *P. falciparum* malaria for a first-line drug in non-HIV patients should be at least 90% and preferably >95% [39]. Various studies have shown that the day 28 parasitological cure rate in non-HIV infected patients with *P. falciparum* treated with AL is >95% [41]. Under the WHO guidelines [39], this study shows an 82.5%, 97.6% and 94.5% ACPR rate by day 28 in the EFV-arm, NVP-arm and control-arm, respectively. Patients in the EFV-arm had a high risk of recurrent parasitaemia and low day 7 lumefantrine plasma concentrations compared to the NVP-arm and the control-arm. Similar results were expected in the NVP-arm and the EFV-arm; however, a better ACPR was observed in the NVP-arm.

The observed results in the EFV-arm may be due to possible DDIs between EFV and lumefantrine during the course of treatment as a result of the induction of common metabolic enzyme CYP3A4, leading to increased clearance of lumefantrine and artemether [28-30,42]. Artemether is responsible for parasite biomass reduction and lumefantrine for cure rate in uncomplicated falciparum malaria [24-26]. Although, NVP and EFV are both reported to be inducers of CYP3A4 and CYP2B6, the induction capacity is reported to be disproportionate. EFV is reported to significantly induce both CYP2B6 and CYP3A4 [29], as opposed to NVP, which strongly induces CYP2B6 than CYP3A4 [43,44]. The differences in the induction capacity of CYP3A4 enzyme might explain the observed differences in the reduction of day 7 lumefantrine plasma concentrations and, ultimately, the ACPR between the two arms.

These results are in agreement with findings from previous studies, which indicated a possible malaria treatment failure in patients treated with AL and EFV [34,45] and better malaria treatment outcome in patients taking AL and NVP [33,34].

Lumefantrine absorption varies considerably among individuals with its bioavailability being improved by fatty meal intake. As this study was unsupervised, poor adherence to treatment or inadequate fatty meal intake might have contributed to the observed sub-optimal day 7 lumefantrine plasma concentrations in the three arms, thus increasing the risk of recurrent parasitaemia. However, an AL cure rate of >96% was recently reported irrespective of whether it was given under supervision, with food or unsupervised [46].

Differences in immune status among the studied population might have also contributed to the observed differences in the ACPR between the three arms. Patients in the EFV-arm had lower mean CD4 cell counts compared with those in the NVP and control-arms, although the differences did not reach statistical significance (P = 0.629). This is in line with previous reports [11,17]. Studies have shown that patients with CD4 cell counts < 300 cell/μl are at high risk of re-infection rather than recrudescence when treated with AL [12,14,47-49]. Because, in the present study, parasite genotyping was not done to distinguish recrudescences from re-infection, the possibility of recrudescence cannot be excluded, in patients with day 7 lumefantrine plasma concentrations below the therapeutic cut-off point [24,25]. Day 7 lumefantrine plasma concentration is a surrogate marker for AUC and lumefantrine AUC correlates well with the treatment response and reflects the degree of exposure of the parasite to lumefantrine after artemether clearance [24,25]. Parasites are more likely to survive and multiply when the drug concentration in a patient’s blood is below the minimum inhibitory concentration to keep down their multiplication rate. The surviving parasites will then re-expand as the drug is eliminated and concentrations fall further, eventually causing a recrudescence [50]. In the present study this cut-off point associated well with RP; seven out of eight patients with recurrent parasitaemia on day 7 had lumefantrine day 7 plasma concentrations below this threshold.

Malaria parasite density was slightly higher among patients in both the NVP-arm and the EFV-arm than in the control-arm, although this difference was not statistically significant. Lower CD4 cell counts in the EFV and NVP-arms than in the control-arm might also have contributed to higher parasite density. This finding is in agreement with other studies, which documented higher parasite density with decreased immunity among HIV-1 infected patients [6,17,46,47]. Additionally, in the present study, patients with recurrent parasitaemia during the 28 days of follow-up had a high baseline parasite count (BPC). Ezzet *et al.* reported high risks of recrudescence in patients with high BPC and low plasma concentration of lumefantrine [51].

While the results presented and discussed above are of clinical importance in improving the quality of life for HIV-1 infected patients on ART with uncomplicated falciparum malaria and treated with AL, nevertheless, they are not without limitations. In the present study, the enrolled patients were carefully screened for fever history, the presence or absence of other obvious causes of fever and the researchers carefully followed up guidelines for malaria screening and diagnosis [20,36], nevertheless, this cannot rule out the inclusion of patients with low asymptomatic *P. falciparum* parasitaemia, while the presented fever was caused by opportunistic infections. Reports from other studies have indicated that the febrile illness that occurs more frequently in HIV/AIDS patients, especially those with low CD4 cell counts, even in the presence of malaria infection, may be caused not by malaria but by other opportunistic infections or may be due to adverse drug reactions, thus complicating the diagnosis of malaria [8,52-54]. Thus, the presence of malaria parasitaemia does not confirm malaria as the sole source of fever in HIV/AIDS patients [52]. Equally, in studies conducted in Uganda and Tanzania reported a rate as low as 4% and 8%, respectively for asymptomatic *P. falciparum* parasitaemia among ART-treated patients and healthy people, respectively [55,56].

Decreasing the risk of false negative malaria, especially in patients with low malaria parasitaemia, using more sensitive diagnostic methods such as the malaria rapid diagnostic test (MRDT) and quantitative real-time polymerase chain reaction (PCR), could have complimented the thick blood smear film method. However, the MRDT and PCR are not without limitations as it is in the case of thick blood film. The accuracy of RDT for the diagnosis of uncomplicated *P. falciparum* infection is reported to be equal or superior to routine microscopy (but inferior to expert microscopy) and do not generate all information provided by microscopy [57]. The thick blood film method used in the current study has been reported to have high specificity and with a sensitivity of up to 4 to 20 parasites/μL of blood with experienced microscopist [58,59].

Thick blood films have been reported to give conflicting results in various studies [60-62]. However, such conflicting results could be minimized by the use of experienced microscopist and increasing examination time/number of microscopic fields examined [63]. In addition, false positive and negative have been reported with the use of MRDT. False negative malaria have been reported in patients with low parasitaemia (<200 parasite/ul), very high parasitaemia (prozone effect), in case of histidine rich protein 2 of *P. falciparum* deficiency (*Pfhrp2* gene deletions) and due to cross-reactions between Plasmodium species. The sensitivity of MRDT has been reported to be reduced to < 75% with < 1000 parasites/μl [57,64,65]. False positive malaria has been reported in patients with other infections, with auto-antibodies (rheumatoid factor), presence of gametocytes and due to persistence of PfHRP2 in the blood stream for a period of up to one month following successful malaria parasite clearance by anti-malarial treatment or self-cleared infection [57,62,64,66]. The PCR has been reported to overestimate malaria [67]. This study involved highly experienced microscopists therefore, the use of thick blood film as solely diagnostic tool might have not adversely affected malaria results. WHO and other guidelines recommend the use of experienced microscopists and light microscope as primary method of malaria diagnosis in endemic areas [36,61,64,68].

In the present study, malaria parasites were not genotyped to differentiate recrudescence from re-infection [39]; in areas of high or moderate malaria transmission, RP are probably due to new infectious bites [35]. Thus, the ACPR (AL effectiveness) reported here might have increased in the three arms studied if these results had been corrected by PCR for re-infection.

In conclusion, the findings of this study suggest that AL is safe and effective in the treatment of uncomplicated falciparum malaria in patients receiving NVP-based ART compared to those receiving EFV-based ART. The results of this study also provide an indication of the possible impact of EFV on the performance of AL and the likelihood of it affecting malaria treatment outcome. Surveillance on the effectiveness and efficacy of AL in HIV infected patients, particularly those on EFV-based ART, should be performed in order to elucidate the role of the latter in the treatment outcome of uncomplicated falciparum malaria.

## Competing interests

The authors declare that they have no competing interests.

## Authors’ contribution

OMSM and SP conceived the study, participated in the study design, coordination, data analysis and manuscript writing. BM participated in study design, data collection and analysis and in the preparation and writing of the manuscript. BN participated in the data analysis as well as manuscript preparation. All authors participated in reading and approving the final manuscript.

## References

[B1] World Health Organization Malaria and HIV Interactions and Their Implications for Public Health Policy[http://whqlibdoc.who.int/publications/2005/9241593350.pdf]

[B2] UNAIDS Joint United Nations Programme on HIV/AIDS Global Report UNAIDS report on the global AIDS epidemic 2012 [http://www.unaids.org/en/media/unaids/contentassets/documents/epidemiology/2012/gr2012/20121120_unaids_global_report_2012_with_annexes_en.pdf]

[B3] Greenwood BM Bojang K Whitty CJ Targett GA Malaria Lancet 2005 365 1487 1498 10.1016/S0140-6736(05)66420-3 15850634

[B4] Cohen C Karstaedt A Frean J Thomas J Govender N Prentice E Dini L Galpin J Crewe-Brown H Increased prevalence of severe malaria in HIV-infected adults in South Africa Clin Infect Dis 2005 41 1631 1637 10.1086/498023 16267737

[B5] Chalwe V Van Geertruyden J Mukwamataba D Menten J Kamalamba J Mulenga M D’Alessandro U Increased risk for severe malaria in HIV-1–infected adults, Zambia Emerg Infect Dis 2009 15 749 755 10.3201/eid1505.081009 19402961 PMC2687012

[B6] Patnaik P Jere CS Miller WC Hoffman IF Wirima J Pendame R Meshnick SR Taylor TE Molyneux ME Kublin JG Effects of HIV-1 serostatus, HIV-1 RNA concentration, and CD4 cell count on the incidence of malaria infection in a cohort of adults in rural Malawi J Infect Dis 2005 192 984 991 10.1086/432730 16107950

[B7] Whitworth J Morgan D Quigley M Smith A Mayanja B Eotu H Omoding N Okongo M Malamba S Ojwiya A Effect of HIV-1 and increasing immunosuppression on malaria parasitaemia and clinical episodes in adults in rural Uganda: a cohort study Lancet 2000 356 1051 1056 10.1016/S0140-6736(00)02727-6 11009139

[B8] Laufer MK van Oosterhout JJ Thesing PC Thumba F Zijlstra EE Graham SM Taylor TE Plowe CV Impact of HIV-associated immunosuppression on malaria infection and disease in Malawi J Infect Dis 2006 193 872 878 10.1086/500245 16479522

[B9] Berg A Patel S Aukrust P David C Gonca M Berg ES Dalen I Langeland N Increased severity and mortality in adults co-infected with malaria and HIV in Maputo, Mozambique: A prospective cross-sectional study PLoS One 2014 9 e88257 10.1371/journal.pone.0088257 24505451 PMC3914956

[B10] Korenromp EL Williams BG de Vlas SJ Gouws E Gilks CF Ghys PD Nahlen BL Malaria attributable to the HIV-1 epidemic, sub-Saharan Africa Emerg Infect Dis 2005 11 1410 1419 10.3201/eid1109.050337 16229771 PMC3310631

[B11] Van Geertruyden JP Mulenga M Chalwe V Michael N Moerman F Mukwamataba D Colebunders R D’alessandro U Impact of HIV-1 infection on the hematological recovery after clinical malaria J Acquir Immune Defic Syndr 2009 50 200 205 10.1097/QAI.0b013e3181900159 19131887

[B12] Hewitt K Steketee R Mwapasa V Whitworth J French N Interactions between HIV and malaria in non-pregnant adults: evidence and implications AIDS 2006 20 1993 2004 10.1097/01.aids.0000247572.95880.92 17053345

[B13] Byakika-Kibwika P Ddumba E Kamya M Effect of HIV-1 infection on malaria treatment outcome in Ugandan patients Afr Health Sci 2007 7 86 92 17594285 10.5555/afhs.2007.7.2.86 PMC1925269

[B14] Kamya MR Gasasira AF Yeka A Bakyaita N Nsobya SL Francis D Rosenthal PJ Dorsey G Havlir D Effect of HIV-1 infection on antimalarial treatment outcomes in Uganda: a population-based study J Infect Dis 2006 193 9 15 10.1086/498577 16323126

[B15] Colebunders R Bahwe Y Nekwei W Ryder R Perriens J Nsimba K Turner A Francis H Lebughe I Van der Stuyft P Incidence of malaria and efficacy of oral quinine in patients recently infected with human immunodeficiency virus in Kinshasa, Zaire J Infect 1990 21 167 173 10.1016/0163-4453(90)91701-E 2230175

[B16] Shah SN Smith EE Obonyo CO Kain KC Bloland PB Slutsker L Hamel MJ HIV immunosuppression and antimalarial efficacy: sulfadoxine-pyrimethamine for the treatment of uncomplicated malaria in HIV-infected adults in Siaya, Kenya J Infect Dis 2006 194 1519 1528 10.1086/508892 17083036

[B17] Van Geertruyden JP Mulenga M Mwananyanda L Chalwe V Moerman F Chilengi R Kasongo W Van Overmeir C Dujardin JC Colebunders R Kestens L D’Alessandro U HIV-1 immune suppression and antimalarial treatment outcome in Zambian adults with uncomplicated malaria J Infect Dis 2006 194 917 925 10.1086/507310 16960779

[B18] Flateau C Le Loup G Pialoux G Consequences of HIV infection on malaria and therapeutic implications: a systematic review Lancet Infect Dis 2011 11 541 556 10.1016/S1473-3099(11)70031-7 21700241

[B19] Francesconi P Fabiani M Dente MG Lukwiya M Okwey R Ouma J Ochakachon R Cian F Declich S HIV, malaria parasites, and acute febrile episodes in Ugandan adults: a case–control study AIDS 2001 15 2445 2450 10.1097/00002030-200112070-00013 11740196

[B20] Birku Y Mekonnen E Bjorkman A Wolday D Delayed clearance of *Plasmodium falciparum* in patients with human immunodeficiency virus co-infection treated with artemisinin Ethiop Med J 2002 40 17 26 12802828

[B21] World Health Organization Guidelines for the Treatment of Malaria, Second Edition[http://www.who.int/malaria/publications/atoz/9789241547925/en/index.html]

[B22] Fehintola FA Scarsi KK Ma Q Parikh S Morse GD Taiwo B Akinola IT Adewole IF Lindegardh N Phakderaj A Ojengbede O Murphy RL Akinyinka OO Aweeka FT Nevirapine-based antiretroviral therapy impacts artesunate and dihydroartemisinin disposition in HIV-Infected Nigerian Adults AIDS Res Treat 2012 2012 703604 22500218 10.1155/2012/703604 PMC3303559

[B23] Djimde A Lefevre G Understanding the pharmacokinetics of Coartem Malar J 2009 8 Suppl 1 S4 10.1186/1475-2875-8-S1-S4 19818171 PMC2760239

[B24] White NJ van Vugt M Ezzet F Clinical pharmacokinetics and pharmacodynamics and pharmacodynamics of artemether-lumefantrine Clin Pharmacokinet 1999 37 105 125 10.2165/00003088-199937020-00002 10496300

[B25] Ezzet F van Vugt M Nosten F Looareesuwan S White NJ Pharmacokinetics and pharmacodynamics of lumefantrine (benflumetol) in acute falciparum malaria Antimicrob Agents Chemother 2000 44 697 704 10.1128/AAC.44.3.697-704.2000 10681341 PMC89749

[B26] Ashley EA Stepniewska K Lindegårdh N McGready R Annerberg A Hutagalung R Singtoroj T Hla G Brockman A Proux S Wilahphaingern J Singhasivanon P White NJ Nosten F Pharmacokinetic study of artemether–lumefantrine given once daily for the treatment of uncomplicated multidrug-resistant falciparum malaria Trop Med Int Health 2007 12 201 208 10.1111/j.1365-3156.2006.01785.x 17300626

[B27] Khoo S Back D Winstanley P The potential for interactions between antimalarial and antiretroviral drugs AIDS 2005 19 995 1005 10.1097/01.aids.0000174445.40379.e0 15958830

[B28] Smith PF DiCenzo R Morse GD Clinical pharmacokinetics of non-nucleoside reverse transcriptase inhibitors Clin Pharmacokinet 2001 40 893 905 10.2165/00003088-200140120-00002 11735608

[B29] de Maat MRM Huitema DRA Mulder WJ Meenhorst LP van Gorp CME Beijnen HJ Population pharmacokinetics of nevirapine in an unselected cohort of HIV-1-infected individuals Br J Clin Pharmacol 2002 54 378 385 10.1046/j.1365-2125.2002.01657.x 12392585 PMC1874435

[B30] Lamson M MacGregor T Riska P Erickson D Maxfield P Rowland L Gigliotti M Robinson P Azzam S Keirns J Nevirapine induces both CYP3A4 and CYP2B6 metabolic pathways Clin Pharmacol Ther 1999 65 137

[B31] German P Parikh S Lawrence J Dorsey G Rosenthal PJ Havlir D Charlebois E Hanpithakpong W Lindegardh N Aweeka FT Lopinavir/ritonavir affects pharmacokinetic exposure of artemether/lumefantrine in HIV-uninfected healthy volunteers J Acquir Immune Defic Syndr 2009 51 424 429 10.1097/QAI.0b013e3181acb4ff 19506482

[B32] German P Greenhouse B Coates C Dorsey G Rosenthal PJ Charlebois E Lindegardh N Havlir D Aweeka FT Hepatotoxicity due to a drug interaction between amodiaquine plus artesunate and efavirenz Clin Infect Dis 2007 44 889 891 10.1086/511882 17304470

[B33] Kredo T Van derWalt JS Mauff K Wiesner L Maartens G Cohen K Smith P Barnes KI Interaction between artemether-lumefantrine and nevirapine-based antiretroviral therapy in HIV-1-infected patients Antimicrob Agents Chemother 2011 55 5616 5623 10.1128/AAC.05265-11 21947399 PMC3232823

[B34] Byakika-Kibwika P Lamorde M Mayito J Nabukeera L Namakula R Mayanja-Kizza H Katabira E Ntale M Pakker N Ryan M Hanpithakpong W Tarning J Lindegardh N de Vries PJ Khoo S Back D Merry C Significant pharmacokinetics interactions between artemether/lumefantrine and efavirenz or in HIV-infected Ugandan adults J Antimicrob Chemother 2012 67 2213 2221 10.1093/jac/dks207 22687893 PMC3465101

[B35] Woodring JV Ogutu B Schnabel D Waitumbi JN Olsen CH Walsh SD Heppner DG Jr Polhemus ME Evaluation of recurrent parasitemia after artemether-lumefantrine treatment for uncomplicated malaria in children in Western Kenya Am J Trop Med Hyg 2010 83 458 464 10.4269/ajtmh.2010.09-0403 20810804 PMC2929035

[B36] Tanzania National Malaria Control Programme National Guidelines for Malaria Diagnosis and Treatment 2006 Dar es Salaam [http://apps.who.int/medicinedocs/documents/s19271en/s19271en.pdf]

[B37] World Health Organization Basic Malaria Microscopy. Part 1 Learner’s Guide 2 [http://whqlibdoc.who.int/publications/2010/9789241547826_eng.pdf]

[B38] Minzi OM Ngaimisi E Shewiyo DH Sasi P Ignace AM Inter-laboratory development and cross validation of a chromatographic method for determination of lumefantrine in human plasma - A proficient capacity assessment of bioanalytical laboratories in East Africa J Anal Bioanal Techniques 2012 3 131 136

[B39] World Health Organization Method for Surveillance of Anti-Malaria Drug Efficacy[http://whqlibdoc.who.int/publications/2009/9789241597531_eng.pdf]

[B40] White JN The assessment of antimalarial efficacy Trends Parasitol 2002 18 458 464 10.1016/S1471-4922(02)02373-5 12377597

[B41] Makanga M Bassat Q Falade CO Premji ZG Krudsood S Hunt P Walter V Beck HP Marrast AC Cousin M Rosenthal PJ Efficacy and safety of artemether-lumefantrine in the treatment of acute, uncomplicated *Plasmodium falciparum* malaria: a pooled analysis Am J Trop Med Hyg 2011 85 793 804 10.4269/ajtmh.2011.11-0069 22049029 PMC3205621

[B42] Hariparsad N Nallani SC Sane RS Buckley DJ Buckley AR Desai PB Induction of CYP3A4 by efavirenz in primary human hepatocytes: comparison with rifampin and phenobarbital J Clin Pharmacol 2004 44 1273 1281 10.1177/0091270004269142 15496645

[B43] Pinzani V Faucherre V Peyriere H Blayac JP Methadone withdrawal symptoms with nevirapine and efavirenz Ann Pharmacother 2000 34 405 407 10917395 10.1345/aph.19156

[B44] Erickson DA Mather G Trager WF Levy RH Keirns JJ Characterization of the in vitro biotransformation of the HIV-1 reverse transcriptase inhibitor nevirapine by human hepatic cytochromes P-450 Drug Metab Dispos 1999 27 1488 1495 10570031

[B45] Huang L Parikh S Rosenthal PJ Lizak P Marzan F Dorsey G Havlir D Aweeka FT Concomitant efavirenz reduces pharmacokinetic exposure to the antimalarial drug artemether-lumefantrine in healthy volunteers J Acquir Immune Defic Syndr 2012 61 310 316 10.1097/QAI.0b013e31826ebb5c 22918158 PMC3511816

[B46] Piola P Fogg C Bajunirwe F Biraro S Grandesso F Ruzagira E Babigumira J Kigozi I Kiguli J Kyomuhendo J Ferradini L Taylor W Checchi F Guthmann JP Supervised versus unsupervised intake of six-dose artemether-lumefantrine for treatment of acute, uncomplicated *Plasmodium falciparum* malaria in Mbarara, Uganda: a randomised trial Lancet 2005 365 1467 1473 10.1016/S0140-6736(05)66416-1 15850630

[B47] Diallo AH Ki-Zerbo G Sawadogo AB Guiguemde TR Severe malaria and HIV in adult patients in Bobo-Dioulasso, Burkina Faso (in French) Med Trop (Mars) 2004 64 345 350 15615384

[B48] Bukirwa H Yeka A Kamya MR Talisuna A Banek K Bakyaita N Rwakimari JB Rosenthal PJ Wabwire-Mangen F Dorsey G Staedke SG Artemisinin combination therapies for treatment of uncomplicated malaria in Uganda PLoS Clin Trials 2006 1 e7 10.1371/journal.pctr.0010007 16871329 PMC1488893

[B49] Kamya MR Yeka A Bukirwa H Lugemwa M Rwakimari JB Staedke SG Talisuna AO Greenhouse B Nosten F Rosenthal PJ Wabwire-Mangen F Dorsey G Artemether-lumefantrine versus dihydroartemisinin piperaquine for treatment of malaria: a randomized trial PLoS Clin Trials 2007 2 e20 10.1371/journal.pctr.0020020 17525792 PMC1876597

[B50] Stepniewska K White NJ Pharmacokinetic determinants of the window of selection for antimalarial drug resistance Antimicrob Agents Chemother 2008 52 1589 1596 10.1128/AAC.00903-07 18299409 PMC2346628

[B51] Ezzet F Mull R Karbwang J Population pharmacokinetics and therapeutic response of CGP 56697 (artemether + benflumetol) in malaria patients Br J Clin Pharmacol 1998 46 553 561 9862244 10.1046/j.1365-2125.1998.00830.x PMC1873796

[B52] Brentlinger PE Behrens CB Kublin JG Challenges in the prevention, diagnosis, and treatment of malaria in human immunodeficiency virus infected adults in sub-Saharan Africa Arch Intern Med 2007 167 1827 1836 10.1001/archinte.167.17.1827 17893303

[B53] Kublin JG Patnaik P Jere CS Miller WC Hoffman IF Chimbiya N Pendame R Taylor TE Molyneux ME Effect of *Plasmodium falciparum* malaria on concentration of HIV-1-RNA in the blood of adults in rural Malawi: a prospective cohort study Lancet 2005 365 233 240 15652606 10.1016/S0140-6736(05)17743-5

[B54] Simooya OO Mwendapole RM Siziya S Fleming AF Relation between falciparum malaria and HIV seropositivity in Ndola, Zambia BMJ 1988 297 30 31 10.1136/bmj.297.6640.30 3044486 PMC1834172

[B55] Nakanjako D Kiragga AN Castelnuovo B Kyabayinze DJ Kamya MR Low prevalence of *Plasmodium falciparum* antigenaemia among asymptomatic HAART treated adults in an urban cohort in Uganda Malar J 2011 10 66 10.1186/1475-2875-10-66 21426579 PMC3071332

[B56] Imperato PJ Malaria parasitemia in healthy Africans in North Mara, Tanzania J Community Health 1986 11 92 97 10.1007/BF01321510 3534007

[B57] Maltha J Gillet P Jacobs J Malaria rapid diagnostic tests in endemic settings Clin Microbiol Infect 2013 19 399 407 10.1111/1469-0691.12151 23438048

[B58] Payne D Use and limitations of light microscopy for diagnosing malaria at the primary health care level Bull World Health Organ 1988 66 621 626 2463112 PMC2491188

[B59] WHO New Perspectives in Malaria Diagnosis Report of a joint WHO/USAID informal consultation, 25–27, October 1999 [http://www.who.int/tdr/publications/documents/malaria-diagnosis.pdf]

[B60] Bejon P Andrews L Hunt-Cooke A Sanderson F Gilbert CS Hill VSA Thick blood film examination for *Plasmodium falciparum* malaria has reduced sensitivity and underestimates parasite density Malar J 2006 5 104 10.1186/1475-2875-5-104 17092336 PMC1636647

[B61] Bowers KM Bell D Chiodini PL Barnwell J Incardona S Yen S Luchavez J Watt H Inter-rater reliability of malaria parasite counts and comparison of methods Malar J 2009 8 267 10.1186/1475-2875-8-267 19939271 PMC2789092

[B62] Kattenberg JH Ochodo EA Boer KR Schallig HD Mens PF Leeflang MM Systematic review and meta-analysis: rapid diagnostic tests versus placental histology, microscopy and PCR for malaria in pregnant women Malar J 2011 10 321 10.1186/1475-2875-10-321 22035448 PMC3228868

[B63] Trape JF Rapid evaluation of malaria parasite density and standardization of thick smear examination for epidemiological investigations Trans R Soc Trop Med Hyg 1985 79 181 184 10.1016/0035-9203(85)90329-3 3890280

[B64] McMorrow ML Masanja MI Abdulla SM Kahigwa E Kachur SP Challenges in routine implementation and quality control of rapid diagnostic tests for malaria–Rufiji District, Tanzania Am J Trop Med Hyg 2008 79 385 390 18784230 PMC5801444

[B65] Huong NM Davis TM Hewitt S Huong NV Uyen TT Nhan DH Cong le D Comparison of three antigen detection methods for diagnosis and therapeutic monitoring of malaria: a field study from southern Vietnam Trop Med Int Health 2002 7 304 308 10.1046/j.1365-3156.2002.00869.x 11952945

[B66] Iqbal J Siddique A Jameel M Hira PR Persistent histidine-rich protein 2, parasite lactate dehydrogenase, and panmalarial antigen reactivity after clearance of *Plasmodium falciparum* monoinfection J Clin Microbiol 2004 42 4237 4241 10.1128/JCM.42.9.4237-4241.2004 15365017 PMC516301

[B67] Srinavasan S Moody A Chiodini PL Comparison of blood-film microscopy, the OptiMAL® dipstick, Rhodamine 123 and PCR for monitoring anti-malarial treatment Ann Trop Med Parasitol 2000 94 227 232 10.1080/00034980050006393 10884866

[B68] WHO Malaria Microscopy Quality Assurance Manual, Version 1 2009 Geneva: World Health Organization

